# Implementation of Neuro-Memristive Synapse for Long-and Short-Term Bio-Synaptic Plasticity

**DOI:** 10.3390/s21020644

**Published:** 2021-01-18

**Authors:** Zubaer I. Mannan, Hyongsuk Kim, Leon Chua

**Affiliations:** 1 Division of Electronics and Information Engineering and Core Research Institute of Intelligent Robots, Jeonbuk National University, Jeonju 567-54896, Korea; zimannan@gmail.com; 2 Division of Electronics Engineering and Core Research Institute of Intelligent Robots, Jeonbuk National University, Jeonju 567-54896, Korea; 3 Department of Electrical Engineering and Computer Sciences, University of California, Berkeley, CA 94720-1770, USA; chua@eecs.berkeley.edu

**Keywords:** long-term potentiation (LTP), long-term depression (LTD), memristor, neuromorphic circuit, short-term facilitation (STF), short-term depression (STD), synaptic plasticity

## Abstract

In this paper, we propose a complex neuro-memristive synapse that exhibits the physiological acts of synaptic potentiation and depression of the human-brain. Specifically, the proposed neuromorphic synapse efficiently imitates the synaptic plasticity, especially long-term potentiation (LTP) and depression (LTD), and short-term facilitation (STF) and depression (STD), phenomena of a biological synapse. Similar to biological synapse, the short- or long-term potentiation (STF and LTP) or depression (STD or LTD) of the memristive synapse are distinguished on the basis of time or repetition of input cycles. The proposed synapse is also designed to exhibit the effect of reuptake and neurotransmitters diffusion processes of a bio-synapse. In addition, it exhibits the distinct bio-realistic attributes, i.e., strong stimulation, exponentially decaying conductance trace of synapse, and voltage dependent synaptic responses, of a neuron. The neuro-memristive synapse is designed in SPICE and its bio-realistic functionalities are demonstrated via various simulations.

## 1. Introduction

Effort is being made to develop a highly technical and specialized artificial intelligence that exhibits human brain-like intelligence. These bioinspired neuromorphic circuits are considered as the new computing platform which outperforms conventional Von-Neumann architectures, as it owns features such as high efficiency, low power consumption, higher adaptability, and enormous parallel processing [1]. Recent technical advances in nano-scale complementary metal oxide semiconductor (CMOS) technologies facilitate researchers to design large-scale neuromorphic circuits utilizing specific very large-scale integration (VLSI) hardware. Additionally, it eases the process of designing complex brain-like intelligence and eventually contributes to connect the human brains directly with machines [2,3]. Recently, researchers developed neuromorphic chips based on CMOS [4,5], subthreshold CMOS [6], OxRAM [7], switched-capacitor (SC) [8], spintronic [9], and memristive [10,11,12,13,14,15] technologies. Among these, memristor is regarded as one of the most potential candidates for designing neuromorphic ICs due to its unique features of bio-synapse, such as operation, low energy consumption, multiple-state operation, impressive scalability, and CMOS compatibility [16].

Inspired with such technological advances, in this literature, we propose a complex neuro-memristive synapse that exhibits the physiological acts of synaptic plasticity of human brain. The proposed memristive synapse impersonates both synaptic potentiation (short-term facilitation (STF) and long-term potentiation (LTP)) and depression (short-term depression (STD) and long-term depression (LTD)) phenomena. Moreover, it is also designed to exhibit the outcome of reuptake and neurotransmitter diffusion processes (i.e., memory fading effect (MFE) in the electronic circuit) of a biological synapse along with the bio-realistic attributes of a neuron. In re-uptake and neurotransmitters diffusion processes, the number of released neurotransmitters of a biological synapse are decreased and eventually result in a decrement of synaptic strength which can be related to the memory fading effect of electronic circuit and also of the human brain.

To accommodate such diverse biological attributes, the neuro-memristive synapse is designed with a composite 1-port of memristor (M) and a controlled capacitor (C_Con_). The memristance and voltage across the memristor act as the artificial synaptic strength and voltage, respectively. The C_Con_ capacitor controls the rate of discharging through memristor (M). In potentiation (active cycle of input stimulation), the composite 1-port is charged whereas the memristor of 1-port is partially or fully discharged in the inactive cycle of stimulation based on the active presence of MFE or depression signals. Similar to biological synapse, the short- or long-term potentiation (STF and LTP) or depression (STD and LTD) of artificial synapse are distinguished based on the time or repetition of input cycle. However, the rate of decrement in synaptic strength for SMF and depression are quite different. Hence, the dissimilar rate of change in synaptic efficacy is designed with two different MOS switches. In addition, after a successive STF or LTP process, the rate of removal of neurotransmitters from synapse for reuptake or diffusion (MFE effect) are quite different. Therefore, separate discharging paths are designed through C_Con_ that facilitate the partial discharging through memristor (M).

The neuromorphic excitatory synapses presented in [4] requires twice more hardware than our proposed synapse to implement only spike-timing dependent plasticity (STDP). The digital-controlled neuromorphic circuit in [5] requires additional circuits to implement synaptic plasticity than the self-sufficient proposed model, hence it is less compatible and area inefficient. The sub-threshold CMOS iono-neuromorphic model in [6] requires a nonvolatile digital storage to store synaptic modification and unable to exhibit short-term synaptic plasticity compared to the proposed memristive synapse. To imitate the STP and LTP phenomena, the proposed neuro-memristive synapse requires only a single memristor unlike the OxRAM synapse in [7] which requires 10 and 20 OxRAM devices, respectively. Moreover, the proposed synapse is bio-realistically more effective, energy and area efficient than the switched-capacitor based conductive synapse in [8] which requires multiple switches, couple of capacitors, and amplifier to implement a single synaptic conductance. The proposed memristive synapse is implemented in circuit with off-the-shelf components unlike the mathematical [11] and macro [12] model of memristive synapse. In addition, the proposed memristive synapse is energy and area efficient compared to CMOS-memristive [13] and excitatory memristive [14] synapses despite of exhibiting more bio-synaptic attributes.

The main advantage of the proposed architecture is that the analog artificial synapse can be utilized in both volatile and nonvolatile configuration with a single memristor, unlike prior literatures which required multiple memory elements. Utilizing the synapse alike operation of memristor, the neuro-memristive synapse can also exhibit the bio-realistic attributes of a neuron along with synaptic plasticity. Since the conductance-based neuromorphic synaptic architecture overcomes the limitations of subthreshold analog- and transistor-based CMOS neural circuits and conventional Von-Neumann architectures, it facilitates to port the proposed design in miniature CMOS ICs.

The neuro-memristive synapse can be used to design spiking neural networks with Hebbian or anti-Hebbian learning algorithms. It can also be utilized in academia to analyze the synaptic functionalities of neuron instead in-vivo or in-vitro analysis, and in future the industrial design might be used to design the human brain-like intelligence.

Rest of the paper is organized as following: the biological background of synaptic transmission and plasticity are described in Section 2 and the working principle of proposed artificial synapse is discussed in Section 3. The simulations and results are presented in Section 4 followed by the concluding remarks in Section 5.

## 2. Neuronal Transmission and Synaptic Plasticity

**Figure 1:**
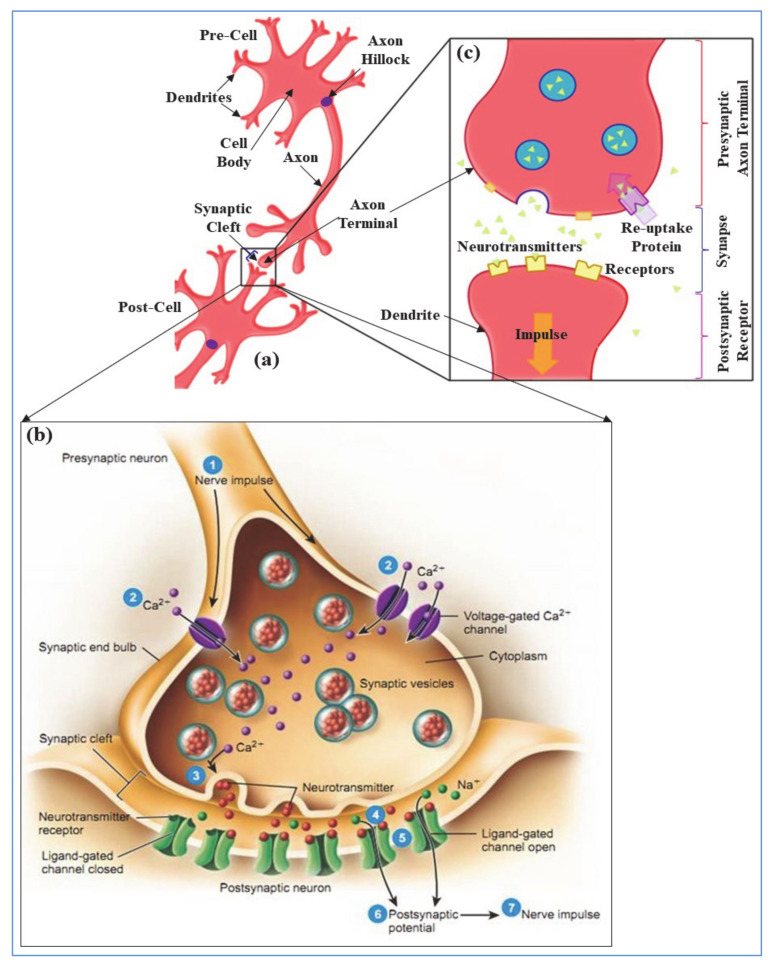
(**a**) Diagram of neuron–neuron communication in a neuronal network, (**b**) steps of bio-synaptic transmission, and (**c**) neurotransmitters reuptake process.

In neurobiology, electrically excitable neurons or cells are transmitting information via synapses. The electro-chemical synaptic transmission among neurons is established when the presynaptic axon terminal (shown in Figure 1a) is depolarized with the arrival of axon-hillock generated action potential (AP) or nerve impulse [17]. Thus, the membrane potential of pre-axon terminal is changed which initiates the opening of voltage gated calcium (Ca^2+^) nano-pore ion channels as shown in Figure 1b. The extracellular Ca^2+^ ions infiltrate to the presynaptic membrane because of steep concentration gradients. Penetrated intracellular Ca^2+^ influx allows the synaptic vesicles to fuse with the presynaptic plasma membrane and release the neurotransmitters in synaptic cleft [18]. The Synaptic cleft, shown in Figure 1b, is a small gap between pre- and post-synaptic neurons (≈20 nm wide) and forms a junction between neurons. The molecules of a released neurotransmitter, in Figure 1b, diffuse across the synaptic cleft and bind to the postsynaptic receptor proteins. Hence, this activates the postsynaptic receptors and leads to the opening or closing of ligand-gated ion channels in post cell. The type of released neurotransmitters have effect on the channel behavior that determines the outcome of postsynaptic cell [19]. For example, acetylcholine (ACh) or glutamate neurotransmitters depolarize (i.e., make the inside of the cell more positive than resting membrane potential) the post-cell and increase the probability of postsynaptic firings. In contrast, GABA or dopamine neurotransmitters hyperpolarize (i.e., make the inside of the cell more negative than resting membrane potential) the post-cell and oppress the post firings. In this paper, we discussed about the excitatory ACh neurotransmitter and its synaptic transmission events. For ACh neurotransmitters, the postsynaptic receptor leads to the opening of sodium (Na^+^) ion channels and the extracellular Na^+^ ions infiltrate into post cell membrane due to concentration gradients. Therefore, the postsynaptic neuron depolarizes, and rises up the cell membrane potential from resting potential, typically −70 mV~−90 mV. When the post membrane potential reaches threshold (typically −55 mV) then it generates an all-or-none electrical impulse (i.e., action potential) and reaches the peak voltage around +40 mV [20]. At peak, the post cell membrane initiates to close Na^+^ ion channels whereas open potassium (K^+^) ion channels (shown in Figure 1b), and intracellular K^+^ ions are moving out of the cell, known as repolarization [21]. The post cell membrane continues to repolarize because of the permeability of K^+^ ion channels and results in an undershoot which is lower than the resting membrane potential, known as hyperpolarization. The membrane potential gradually recovers the undershoot and stabilizes to resting membrane potential [17].

Generation of postsynaptic action potentials in chemical synaptic transmission is proportional to the probability and pattern of neurotransmitter release, and the receptor sensitization of postsynaptic neuron [22,23]. In contrast, after successive transmission of a neural impulse, reuptake process reabsorbs the diffused neurotransmitters from synaptic cleft through a neurotransmitter transporter and brings back to pre-cell membrane as shown in Figure 1c. Reuptake allows the recycling of neurotransmitters and regulates the level of presence of neurotransmitter in synapse which is essential for normal synaptic physiology. Thereby, it controls the lasting durability of released neurotransmitters that results from an excitation [24]. However, in the diffusion process neurotransmitters are detached from the post receptors, drifting out of the synaptic cleft by breaking down with specific enzymes, and absorbed by pre-glial cells for resynthesizing to new neurotransmitters. Both the reuptake and diffusion process decreases the synaptic efficacy of a successive neuronal communication by removing the chemical messenger (i.e., neurotransmitters) from the synaptic cleft [19]. This self-removal mechanism of the neurotransmitters after successive transmissions of a neural impulse can be related to the memory fading effect in the electronic circuit.

Synaptic plasticity is the ability of a synapse to modify its strength or efficacy over a certain period of time. Depending upon the durability of synaptic modification, plasticity is categorized in two types: short-term plasticity and long-term plasticity. Short-term plasticity lasts only for a couple of minutes or less whereas long-term plasticity persists for hours, months or even years [19]. Moreover, based on synaptic modification, short- and long-term plasticity are classified as: short-term potentiation or facilitation (STF) and depression (STD), and long-term potentiation (LTP) and depression (LTD). Both STF and LTP strengthen the synapse in accordance with the perishable release of presynaptic neurotransmitters. Contrarily, STD and LTD weaken the synapse by blocking the neurotransmitters release in spite the presence of presynaptic stimulus, and remove the neurotransmitters from synaptic cleft [19].

STF initiates with close successive presynaptic stimulations that release a higher amount of neurotransmitters, and swiftly strengthen the synapse for a shorter period of time [25]. Unlike STF, LTP persistently strengthens the synapse depending upon the recent patterns of synaptic activity. It produces a long-lasting synaptic enhancement between two neurons by secreting an enormous number of neurotransmitters in the synaptic cleft [26]. Both STF and LTP are considered as one of the major neuromorphic foundations of learning and memory in neuronal communication, as memories are thought to be encoded by the modification of synaptic strength [27].

STD induces with the depletion of neurotransmitter vesicles despite the presence of presynaptic stimulations and weakens the synaptic efficacy (i.e., strength) over a short period of time [25]. However, LTD refers to an activity-dependent reduction in the synaptic efficacy that lasts hours or longer based on the patterns of stimulation. It selectively weakens the synapses disregard of presynaptic stimulation, and builds a productive use of synaptic strengthening caused by LTP. Moreover, it facilitates encoding of new information by stabilizing the neuronal circuit [27].

## 3. Artificial Neuro-Memristive Synapse

**Figure 2:**
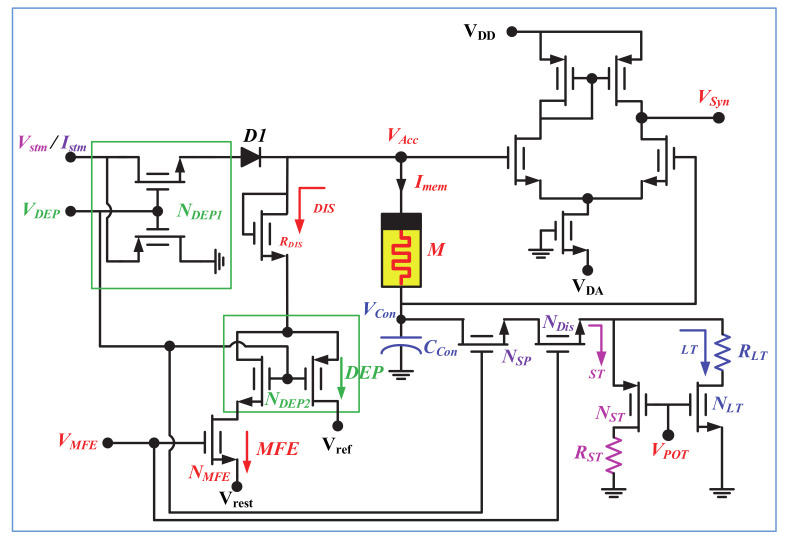
Proposed neuro-memristive synapse.

The neuro-memristive synapse, shown in Figure 2, is designed with a composite 1-port of memristor and capacitor to incorporate the bio-diverse attributes of synaptic plasticity. Memristor [28] is used to imitate the bio-realistic functionalities of a synapse whereas capacitor is utilized to control the partial or full discharging through memristor (M). A depression switch (*N_DEP1_*) is used to regulate the input of memristor where diode (D1) is used to block the reverse current. N_DEP1_ decides between the potentiation (*V_DEP_* = high) or depression (*V_DEP_* = low) mode of operation of the proposed memristive synapse and controls the flowing of input current (*I_stm_*) through the composite 1-port which is defined as:

(1) $$I_{mem}\;=\;\left\{\begin{array}{c} I_{stm},for\;V_{DEP}>0\;\left(potentiation\right)\\ 0,for\;V_{DEP}<0\;\left(depression\right)\;\; \end{array}\right.$$

The memristance of the memristor can be determined as:

(2) $$M_{syn}\;=\;\frac{V_{Acc}\;-V_{Ccon}}{I_{mem}}=M\left(0\right)+V_{CT}R_{T},$$

where *M*(0) = *R_s_* is the initial state, and *V_CT_* and *R_T_* are the intrinsic parameters of the memristor, respectively (Section S1 of Supplementary Materials).

The volatile memory fading effect (i.e., neurotransmitter diffusion or reuptake processes of bio-synapse) is designed with a discharging path through MOS resistor R_DIS_. The synaptic weakening process of a bio-synapse for reuptake or diffusion, and depression mechanisms are quite dissimilar and could not occur at once. Thus, two separate discharging paths are included through R_DIS_ resistor which is controlled by a secondary depression switch N_DEP2_ and memory fading switch N_MFE_. For a high V_MFE_ and V_DEP_, the neuro-memristive synapse will operate in MFE mode and the memristor (M) will discharge through N_MFE_ switch (as shown with red arrowhead). Contrarily, for low V_DEP_, the proposed memristive synapse will exhibit depression irrespective of V_MFE_, and discharges through N_DEP2_ switch (as shown with green arrowhead). Moreover, according to biology, the rate of removal of neurotransmitters from synaptic cleft slows down with high frequency repetitive or rhythmic stimulations (i.e., STF or LTP) that results in slower weakening of bio-synapse. Therefore, the synaptic weakening process of a LTP exhibiting synapse is the slowest than that of STF and normal synaptic response (NSR) exhibiting synapse. This dynamic synaptic weakening attributes of synapse is incorporated with a separated discharge path through control capacitor C_Con_. The discharging path is activated in the inactive cycle of input (i.e., active period of V_MFE_) and further regulates with synaptic plasticity switch N_SP_. When V_MFE_ = high and V_DEP_ = high (i.e., no synaptic depression), the capacitor (C_Con_) will partially discharge through memristor and mostly discharge through N_ST_ or N_LT_ switch depending upon V_POT_. The rate of discharging through N_LT_ is higher than N_ST_. For example, if the neuro-memristive synapse exhibits LTP in active cycle and MFE in inactive cycle with V_DEP_ = high, then the C_Con_ capacitor mostly discharge through N_LT_ (with high V_POT_) and partially discharge through memristor. Hence, the neuro-memristive synapse will exhibit dissimilar synaptic weakening (i.e., dissimilar rate of removal of neurotransmitters) depending on NSR, STF, and LTP exhibiting phenomena. However, for V_MFE_ = high and V_DEP_ = low (i.e., active presence of synaptic depression), the C_Con_ capacitor will fully discharge through memristor and there exists no discharging through N_ST_ or N_LT_. In addition, the memristive synapse can be operated in nonvolatile configuration by oppressing the V_MFE_ and V_DEP_ signals. Output of the proposed synapse (V_Syn_) is obtained across the memristor using a CMOS differential amplifier and determined as:

(3) $$V_{syn}\;=\;V_{Acc}\;-V_{Ccon},$$

where the biasing current of CMOS amplifier depends on the input stimulation. The proposed synapse is operable with both current and voltage input, however, the optimal performance is achieved with current stimulation (Section S2 of the Supplementary Materials). For voltage stimulation, the reference voltage of CMOS amplifier V_DA_ = −V_in_ whereas for current stimulation V_DA_ = −I_in_ × (R_L_ = 10 KΩ). The operating modes of proposed neuro-memristive synapse is included in Table 1.

## 4. Results and Simulations

The bio-realistic features of the proposed neuro-memristive synapse are verified with various SPICE simulations. Due to the commercial unavailability of memristor, a realistic and diverse memristor emulator [28] is used to design the neuro-memristive synapse. To avoid the unintended consequences of nonlinear ionic dopant drift (Section S1 of Supplementary Materials) at boundaries of a memristor [29,30,31], we follow the common practice of initializing the memristance of a memristor in a linear region (2 KΩ~14 KΩ) [32,33]. Therefore, we initialized our memristor emulator at M(0) = 2 KΩ and limit its operation within the linear region. The circuit parameters used to obtain the experimental results are enlisted in Table 2.

## 4.1. Normal Synaptic Response (NSR)

**Figure 3:**
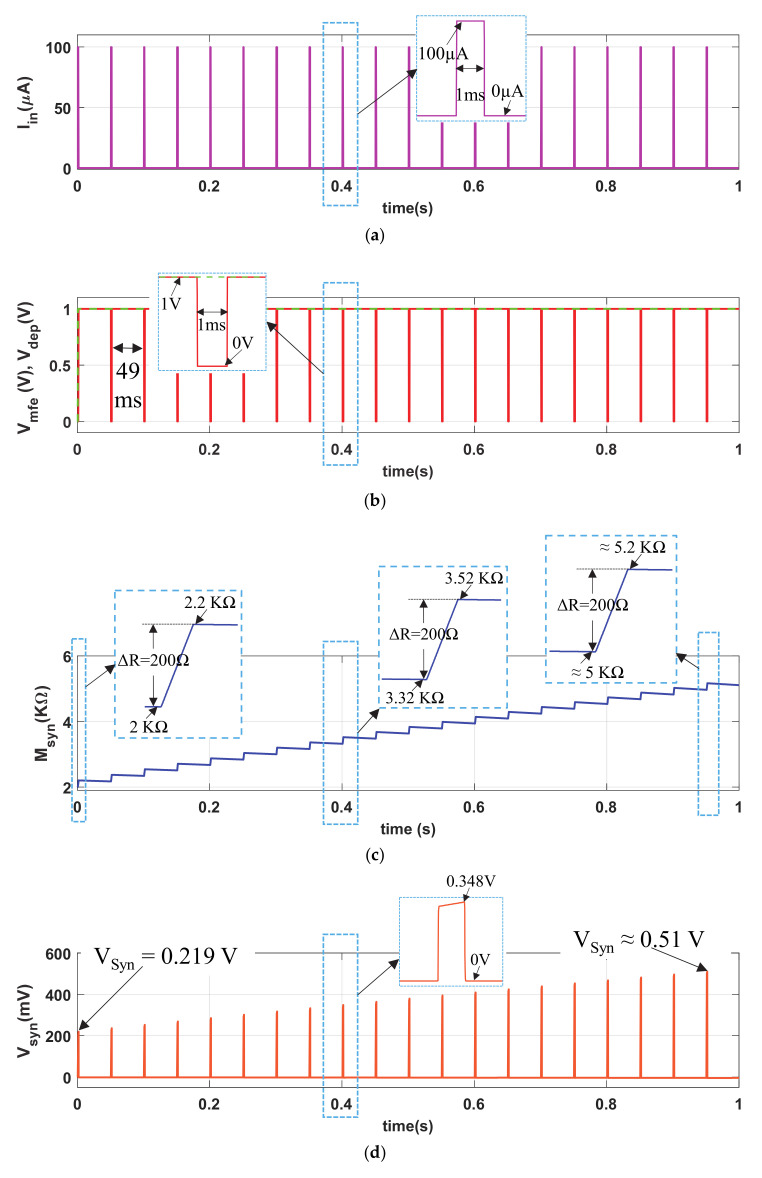
Normal synaptic response (NSR) of the proposed memristive synapse: (**a**) current stimulus (I_in_), (**b**) memory fading effect (V_mfe_) and depression (V_DEP_) signals, artificial (**c**) synaptic strength (M_syn_), and (**d**) synaptic voltage (V_syn_).

The normal synaptic response (NSR) is demonstrated with a pulse current input I_in_ (pulse amplitude PA = 100 µA, pulse width PW = 1 ms and pulse period PP = 50 ms) with a duty ratio of 2%, shown in Figure 3a. We chose the pulse width PW = 1 ms as the duration of biological action potential is typically 1 ms~3 ms. The circuit response of the proposed neuro-memristive synapse for shorter (PW = 0.25 ms) and lengthier (PW = 1.5 ms) pulse width are shown in Section 3 of the Supplementary Materials. The 1′ s complement memory fading signal (V_mfe_, red curve) and the depression signal (V_DEP_, green dotted curve) are shown in Figure 3b. Figure 3c shows the artificial synaptic strength (M_syn_) of the neuro-memristive synapse. Observe from Figure 3c and its inset that the memristance of memristor (M) linearly increase about ΔR = 200 Ω in each cycle of an input stimulation I_in_ = 100 µA. However, the memristance gradually decreases in the active cycle of V_mfe_ (i.e., inactive cycle of I_in_). Such linear increment or decrement in memristance is observed because of our choice of operating the memristor in linear region. It is also observed from Figure 3c that the buildup in memristance is faster than the rate of decrement, which is qualitatively similar to that of a biological synapse. Due to the higher cyclic buildup in M_syn_, the artificial synaptic voltage V_syn_, shown in Figure 3d, increases monotonically.

Figure 3 shows that the proposed neuro-memristive synapse cyclically increases its synaptic efficacy for a non-neutral stimulation (a biological synapse does not modify its synaptic strength at neutral stimulation—δ, θ, α-bands of brainwaves), and similar to a biological synapse it eventually boosts up the probability of postsynaptic firings.

## 4.2. Long-Term Synaptic Plasticity

**Figure 4:**
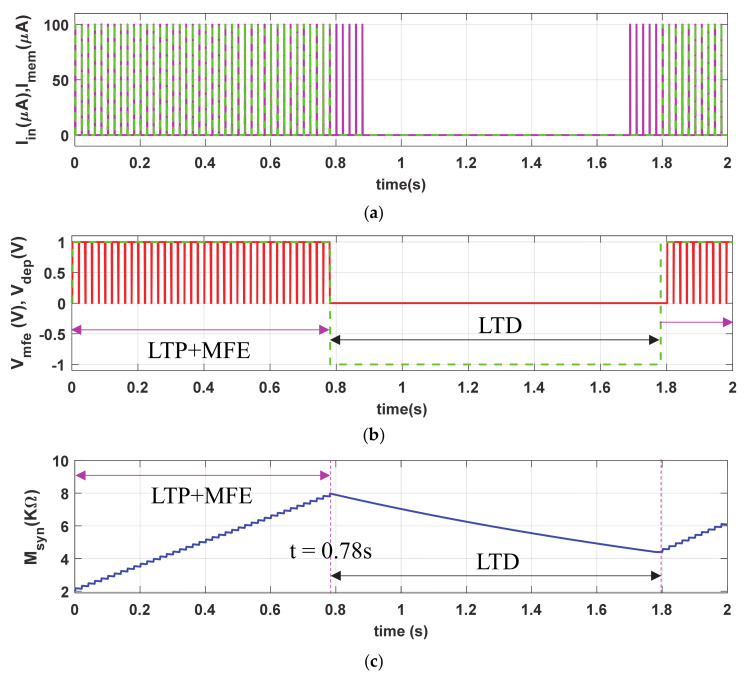

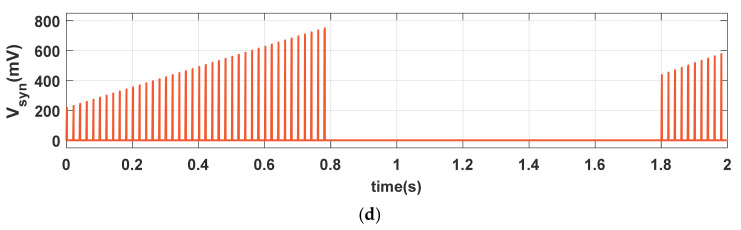
Long-term potentiation (LTP) and long-term depression (LTD) synaptic response of neuro-memristive synapse: (**a**) input stimulus (I_in_) and current stimulus passes through proposed synapse (I_mem_), (**b**) control signals of V_mfe_ and V_DEP_, artificial synaptic (**c**) strength (M_syn_), and (**d**) voltage (V_syn_).

To demonstrate the long-term plasticity (LTP and LTD) phenomena, we stimulated the proposed synapse with same I_in_ (PA = 100 µA and PW = 1 ms), shown with magenta solid curve in Figure 4a, but with more than twice duty ratio (PP = 20 ms) than that of Figure 3a. The dotted green curve (I_mem_), in Figure 4b, shows the cyclic current stimulation passes through the neuro-memristive synapse which depends on the depression input (i.e., V_DEP_ = high). Figure 4b shows the depression and MFE signals. The synaptic efficacy (M_syn_), shown in Figure 4c, gradually increases with each stimulation in LTP period (t ≤ 0.78 s) despite the presence of active V_mfe_. However, it monotonically decreases in the LTD period (0.78 s ≤ t ≤ 1.8 s) in spite the presence of presynaptic stimulation I_in_ (shown in Figure 4a). During the LTD period, the memristive synapse is unable to return to initial state M(0) = 2 KΩ because of the higher buildup in synaptic efficacy in LTP period. Hence, the proposed synapse produces higher V_syn_ (shown in Figure 4d) when the presynaptic stimulations return to the active state after t ≥ 1.8 s than the initial period (t < 0.1 s).

The neuro-memristive synapse effectively impersonates the long-term plasticity phenomena as it produces a long-lasting enhancement in the synaptic efficacy both in the LTP and LTD processes (shown in Figure 4) and results in higher possibilities of postsynaptic firings with the return of input stimulations. Such long lasting synaptic enhancement can also be seen in biological synapse.

## 4.3. Short-Term Synaptic Plasticity

**Figure 5:**
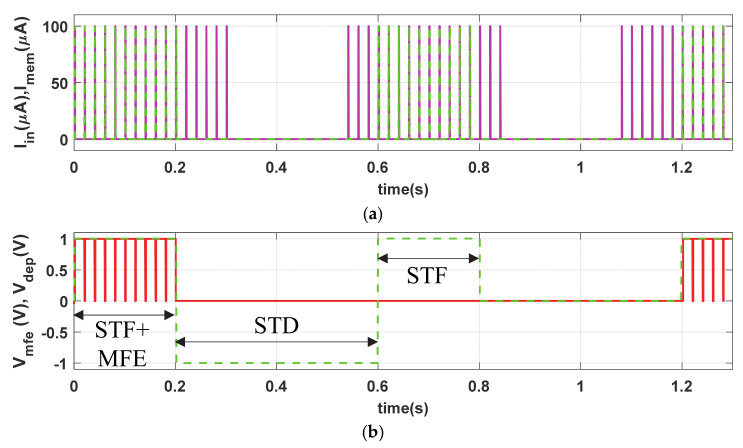

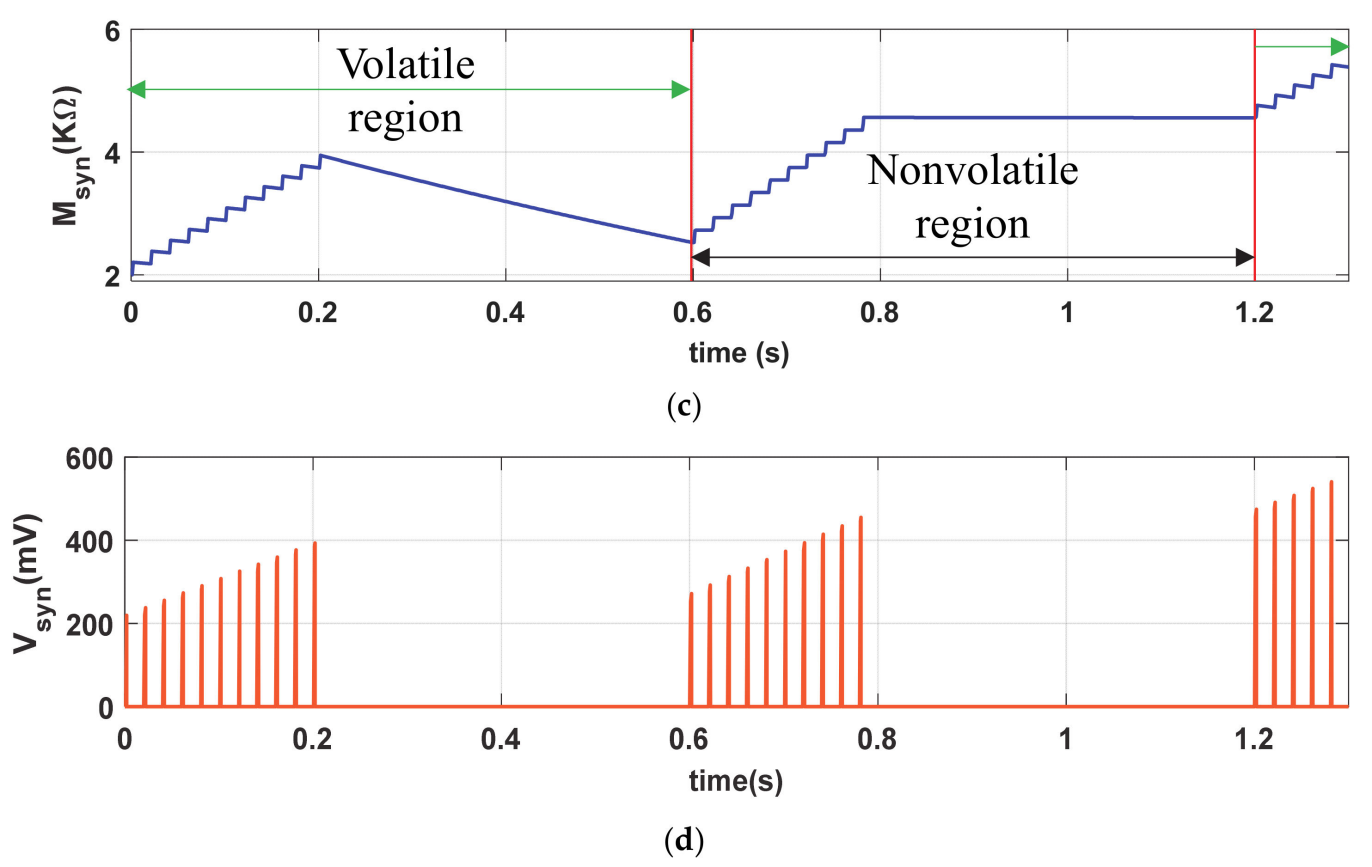
Volatile short-term facilitation (STF) and short-term depression STD, and nonvolatile synaptic responses of the proposed circuit. (**a**) Input stimulus (I_in_) and current stimulus passes through proposed synapse (I_mem_), (**b**) V_mfe_ and V_DEP_ signals, artificial synaptic (**c**) efficacy (M_syn_), and (**d**) voltage (V_syn_).

The short-term plasticity (STF and STD) phenomena of the proposed memristive synapse is demonstrated by providing a verity of diverse stimulation conditions. The input stimulus I_in_ (PA = 100 µA, PW = 1 ms and PP = 20 ms), shown in Figure 5a, remains the same as LTP but the stimulation time period for STF is limited to t = 0.2 s, as shown in Figure 5b. The close successive stimulus assists in gradually accumulation of synaptic efficacy, in Figure 5c, irrespective of the presence of V_mfe_. In contrast, the proposed artificial synapse gradually decreases its synaptic efficacy in the active period of STD in a similar passion to that of volatile bio-synapse. However, to demonstrate the non-volatility, we provide I_in_ stimulations in the proposed synapse in absence of V_mfe_ (0.6 s ≤ t ≤ 1.2 s) as shown in Figure 5b. Observe from Figure 5c that the synaptic strength is only increased (for 0.6 s ≤ t ≤ 1.2 s) in absence of V_mfe_ and active presence of STF (0.6 s ≤ t ≤ 0.8 s). It holds the synaptic strength when the STF is withdrawn during 0.6 s ≤ t ≤ 0.8 s. However, the synaptic efficacy increases and decreases in presence of both STF (V_DEP_ = High) and V_mfe_ at t ≥ 1.2 s and produces higher synaptic voltage (V_syn_) compared to t ≤ 0.1 s, shown in Figure 5d, due to higher buildup in M_syn_.

Similar to bio-synapse, the strength of artificial neuro-memristive synapse (M_syn_) increases swiftly in STF due to close successive stimulus and decreases monotonically in STD where the higher buildup in synaptic efficacy eases the postsynaptic firings.

**Figure 6:**
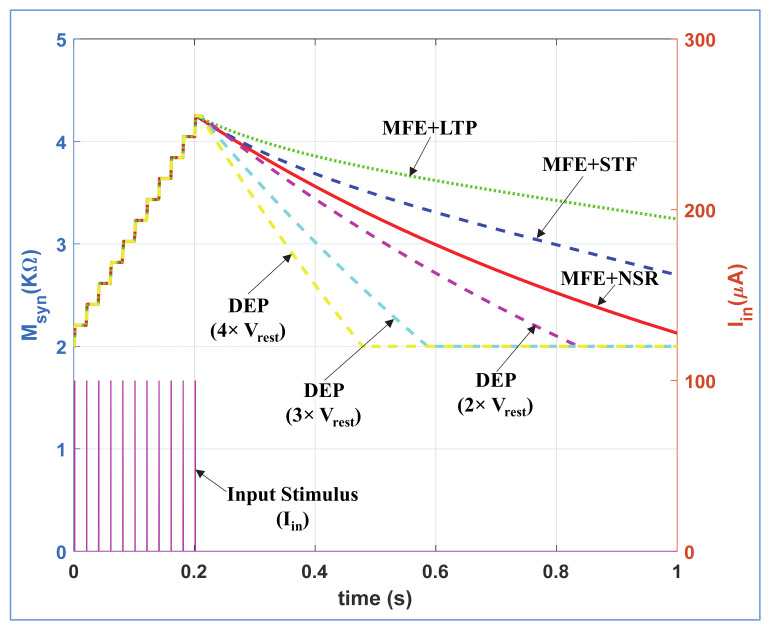
Comparison of synaptic weakening (M_syn_) between the different modes of proposed neuro-memristive synapse.

According to neuro-biology, the rate of removal of neurotransmitters from synaptic cleft (i.e., synaptic weakening) are dissimilar for depression, STF, LTP, and NSR mechanisms. To demonstrate this phenomenon, we stimulated the NSR, STF, LTP, and depression modes of the proposed neuromorphic synapse with same stimulus I_in_ (PA = 100 µA, PW = 1 ms and PP = 20 ms) for t ≤ 0.2 s as shown in Figure 6. In NSR, STF and LTP modes, we provide V_mfe_ = 0 for t ≤ 0.2 s, and V_mfe_ = high for t ≥ 0.2 s where V_DEP_ = high remains same for all time. Contrarily, in depression mode, we provide V_DEP_ = high for t ≤ 0.2 s and V_DEP_ = low for t ≥ 0.2 s where the V_mfe_ = 0 remains same for all time. We provide the same V_POT_ signals for STF and LTP as enlisted in Table 1. Figure 6 shows that the swift artificial synaptic weakening happens in depression mode compared to NSR, STF, and LTP modes with active V_mfe_. In LTP mode (with active V_mfe_), the synaptic strength decreases more slowly than in STF and NSR. Moreover, with different V_ref_ = m*V_rest_ (shown in Figure 2), the rate of change in synaptic efficacy is controllable for depression mode as shown with cyan and magenta dotted curves in Figure 6. We incorporate this dynamic depression option in our design as the rate of removal of neurotransmitters from synaptic cleft for depression mechanism might varied with different types of bio-synapses. The synaptic weakening response of the proposed neuro-memristive synapse is qualitatively equivalent to the attributes of biological synapse.

## 4.4. Strong Stimulation Response

**Figure 7:**
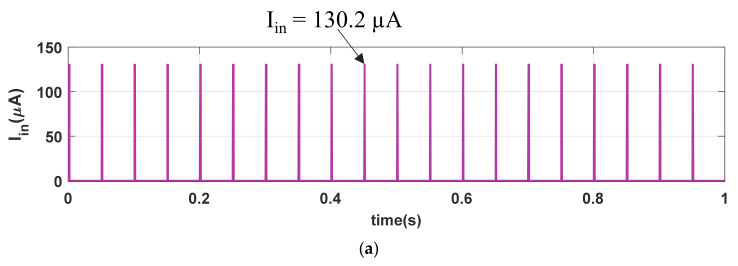

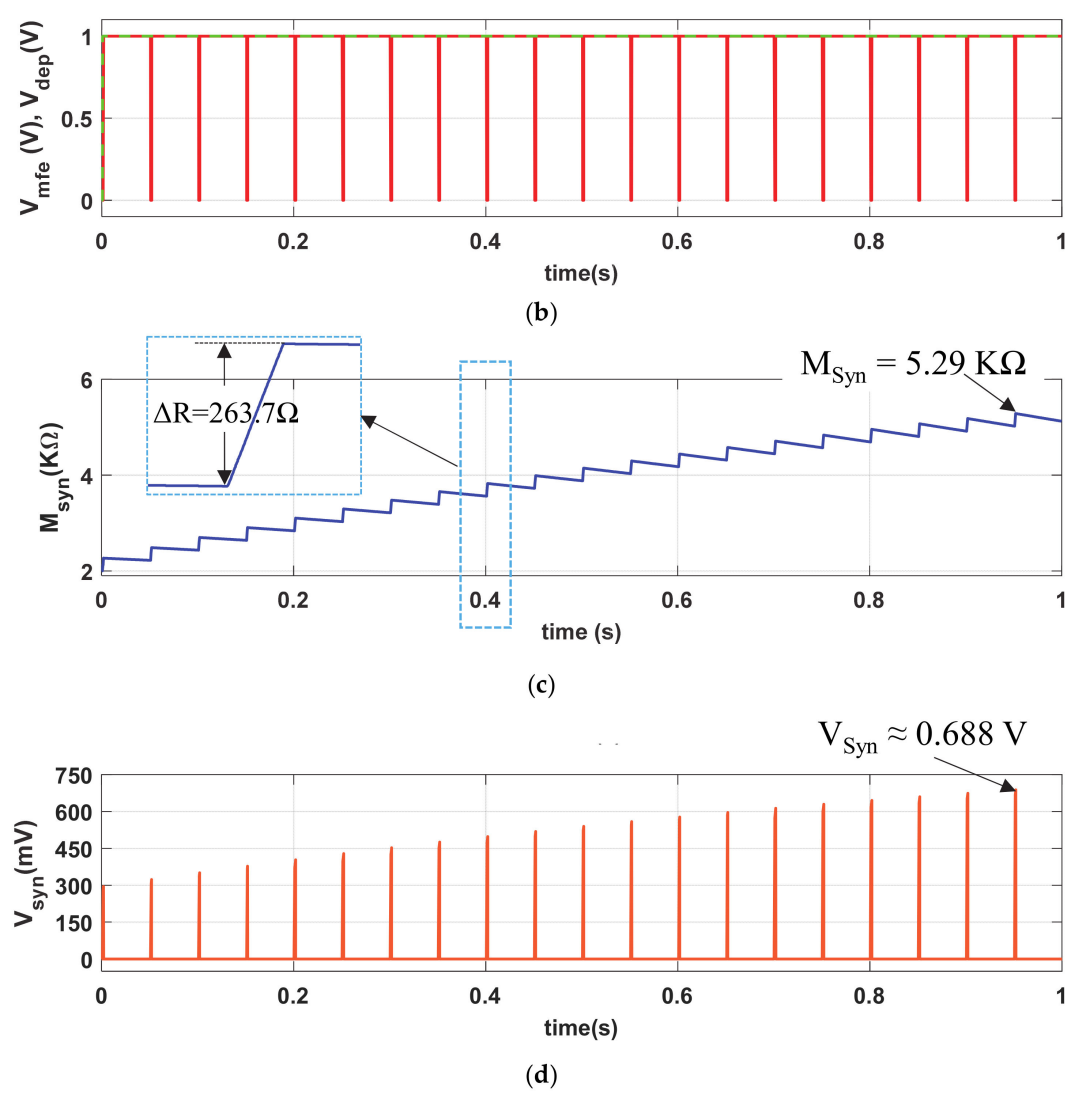
Strong stimulation response of neuro-memristive synapse. (**a**) Strong stimulus (I_in_), (**b**) V_mfe_ and V_DEP_ signals, artificial (**c**) synaptic strength (M_syn_), and (**d**) synaptic voltage (V_syn_).

We further investigate the strong stimulating phenomenon in our memristive synapse with I_in_ (PA = 130.2 µA, PW = 1 ms, and PP = 50 ms) as shown in Figure 7a. Observe that the stimulating inputs in Figure 3a and Figure 7a and are indifferent except the pulse amplitude (PA). Figure 7b shows the similar memory fading (V_mfe_) and depression (V_dep_) signals to that of Figure 3b. Due to the strong stimulus, the memristive synapse accumulates higher synaptic strength, shown in inset of Figure 7c, in each cycle of stimulation than that of M_syn_ in Figure 3c. Hence, the produced synaptic voltage (V_syn_), shown in Figure 7d, is also greater than that of in Figure 3d. The artificial synapse with strong-stimulation exhibits stronger enhancement in its synaptic strength than the regular stimulation and increase the possibilities of probable postsynaptic firings. Therefore, the neuro-memristive synapse effectively imitates the strong stimulation phenomenon of bio-synapse.

The above simulation results of different synaptic modes show that the proposed synaptic architecture efficiently impersonates the neuro-physiological acts of bio-synapse. Moreover, Table 3 shows the power consumption of the neuro-memristive synapse for different synaptic modes. Observe that higher power is consumed by the neuro-memristive synapse in long-term plasticity and strong stimulation modes whereas the lower power consumption occurs with short-term plasticity modes. The power consumption of the proposed memristive synapse is similar to the energy consumption of biological neuron as it requires a significant amount of power to process the information for long-term synaptic plasticity and strong stimulation.

## 5. Concluding Remarks

In this paper, we proposed a neuro-memristive synapse that impersonate the bio-realistic attributes of synapse and its plasticity. The impersonating features of proposed artificial synapse is verified with various SPICE simulation vis-à-vis biological phenomena. The memristive synapse explicitly exhibits the short-term (STF and STD) and long-term (LTP and LTD) plasticity phenomena. The neurotransmitter removal phenomenon through reuptake and diffusion mechanism is also exhibited with the proposed synapse. In addition, it reveals the bio-realistic behaviors, especially strong stimulation, exponentially decaying conductance trace, and voltage dependent synaptic responses, of a neuron.

Unlike multiple memory elements based prior artificial synapse, the proposed neuromorphic circuit is implemented in circuit with off-the-shelf devices and can be utilized in both volatile and nonvolatile configuration. It is not just another synaptic model based only on mathematical or macro-models of memristor. Since the conductive neuromorphic architectures overcomes the limitations of subthreshold analog- and transistor-based CMOS neural circuits and conventional Von-Neumann architectures, hence, the proposed bio-inspired synapse can be port in miniature CMOS ICs. The neuro-memristive synapse can be used to design spiking-neural networks with Hebbian or anti-Hebbian learnings. It can also be utilized in academia to analyze the synaptic functionalities of neuron instead in-vivo or in-vitro analysis and hopefully the industrial design might be used in future to design the human brain-like intelligence.

## Figures and Tables

**Table 1:** Modes of operation of the proposed neuro-memristive synapse.

Memory Type	Synaptic Mode	Synaptic Response	Operating Signals		
			V_DEP_	V_MFE_	V_POT_
Volatile	Potentiation	NSR + MFE	V_H_	V_H_	0
		STF + MFE	V_H_	V_H_	V_L_
		LTP + MFE	V_H_	V_H_	V_H_
	Depression	STD	V_L_	V_L_	V_H_/V_L_
		LTD	V_H_	V_H_	V_H_/V_L_
Nonvolatile	Potentiation	NSR	V_H_	V_L_	0
		STF	V_H_	V_L_	0
		LTP	V_H_	V_L_	0

**Table 2:** Circuit parameters of the neuro-memristive synapse that used in experiments.

Parameters	Value	Parameters	Value
V_DD_/V_H_	3 V	V_DA_	−V_in_
V_SS_/V_L_	−3 V	C_Con_	15 µF
V_rest_	−90 mV	R_ST_	2 KΩ
V_ref_	4 × Vrest	R_LT_	1 KΩ
V_POT_	0/VH/VL	R_L_	10 KΩ

**Table 3:** Power consumption of different synaptic modes of the neuro-memristive synapse.

Synaptic Modes	Power Consumptions (µW)
Normal Synaptic Response (NSR)	196.553
Short-term Plasticity volatile region (STF + STD)	131.994
Short-term Plasticity Nonvolatile region (STF)	156.83
Long-term Plasticity	452.95
Strong Stimulation	424.921

## Data Availability

No Data available online. For further query email to corresponding author (hskim@jbnu.ac.kr).

## Supplementary Materials

Supplementary material is provided. The following are available online at https://www.mdpi.com/1424-8220/21/2/644/s1, Figure S1: Circuit diagram of the incremental memristor emulator that used to design the neuro-memristive synapse, Figure S2: (**a**) Relationship between memristance vs. time for the linear and nonlinear models (Joglekar’s nonlinear ionic-drift [29]) of the TiO2 memristors, (**b**) memristance vs. time relationship of the incremental and decremental memristor emulator, Figure S3: Frequency-dependent pinched hysteresis loops for zero-mean periodic stimulation of Iin = 100µA for (**a**) bi-polar sinusoidal input, and (**b**) pulse input with frequencies f = 10 Hz, 20 Hz, 50 Hz, 100 Hz, and 800 Hz, Figure S4: Normal synaptic response of the proposed memristive synapse for a given voltage stimulation: (**a**) voltage stimulation (Vin = 0.75 V), (**b**) memory fading effect (Vmfe) and depression (VDEP) signals, (**c**) current passing through the memristor emulator of neuro-memristive synapse, (**d**) artificial synaptic strength (Msyn), and (**e**) synaptic voltage (Vsyn), Figure S5: Normal synaptic response of the proposed neuro-memristive synapse with shorter pulse width: (**a**) current stimulus (Iin), (**b**) memory fading effect (Vmfe) and depression (VDEP) signals, artificial (**c**) synaptic strength (Msyn), and (**d**) synaptic voltage (Vsyn), Figure S6: Normal synaptic response of the proposed neuro-memristive synapse with lengthier pulse width: (**a**) current stimulus (Iin), (**b**) memory fading effect (Vmfe) and depression (VDEP) signals, artificial (**c**) synaptic strength (Msyn), and (**d**) synaptic voltage (Vsyn), Table S1. Equations of hp TiO2 Memristor and Memristor Emulator.
