# Supplementary material for: Implementation of Neuro-Memristive Synapse for Long-and Short-Term Bio-Synaptic Plasticity
Source: Sensors (Basel). 2021 Jan 18;21(2):644. doi: 10.3390/s21020644 (PMC7831501; doi:10.3390/s21020644)
Supplement: Supplementary file 1 [file sensors-21-00644-s001.pdf]

sensors

MDPI

Supplementary Materials

Implementation of Neuro-memristive Synapse for Long- and Short- term Bio-synaptic Plasticity

Zubaer I. Mannan[1], Hyongsuk Kim [2,*] and Leon Chua [3]

[1] Division of Electronics and Information Engineering and Core Research Institute of Intelligent Robots, Jeonbuk National University, Jeonju 567-54896, Republic of Korea; zimannan@gmail.com
[2] Division of Electronics Engineering and Core Research Institute of Intelligent Robots, Jeonbuk National University, Jeonju 567-54896, Republic of Korea; hskim@jbnu.ac.kr
[3] Department of Electrical Engineering and Computer Sciences, University of California, Berkeley, CA 94720-1770, USA; chua@eecs.berkeley.edu

* Correspondence: hskim@jbnu.ac.kr;

Supplementary Materials

Section 1. Memristor Emulator

To design the neuro-memristive synapse, we used the incremental type (i.e., resistance changes from low resistive state to high resistive state) of memristor emulator presented in [28]. The memristor emulator, shown in Figure S1, is designed based on *hp* TiO_2 memristor and exhibits the similar behavioral attributes. Table S1 shows the defining equations of *hp* TiO_2 memristor and the memristor emulator in [28].

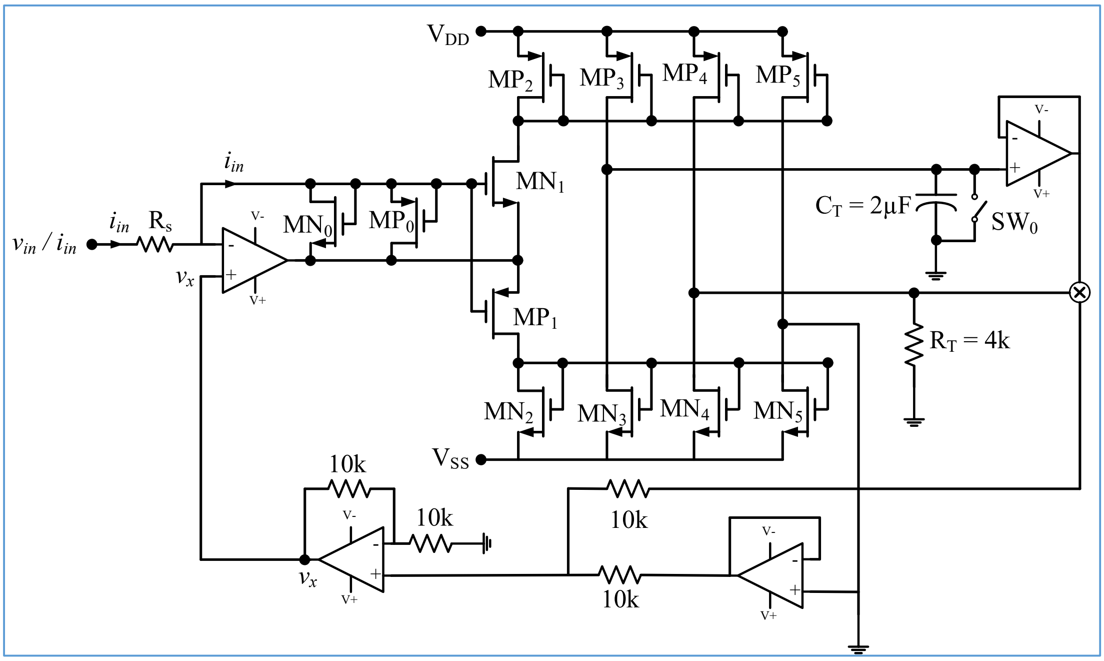

Copyright: © 2021 by the authors. Licensee MDPI, Basel, Switzerland. This article is an open access article distributed under the terms and conditions of the Creative Commons Attribution (CC BY) license (http://creativecommons.org/licenses/by/4.0/).

Figure S1. Circuit diagram of the incremental memristor emulator that used to design the neuro-memristive synapse.

However, the ideal equations of TiO_2 memristor exhibits linear ionic-dopant drift whereas the fabricated device shows nonlinear ionic-dopant drift features. The Joglekar's window function [29] best fits and perfectly analyzed the nonlinear ionic-dopant drift effects presented at the boundaries (w = 0 and w = D) of TiO_2 memristor. Figure S2a shows that the relation between memristance vs. time (i.e., ultimately memristance vs. charge) for the linear and nonlinear models [29] of TiO2 memristors where positive integer p represents the power of Joglekar's window function ($F_P(x) = 1 -(2x-1)^{2P}$), where x is state variable. The nonlinear ionic dopant drift of the memristor creates unintended consequences (i.e., erroneous weight programing in neural networks) and to avoid such consequences it is a common practice to initialize the memristor somewhere in the linear region of its memristance range [32,33]. The Joglekar window function, in Figure S2a, shows that even for the highly nonlinear case of p = 1, the memristance vs. time curve exhibits linearity of operation over the range from 2K to 14K. Moreover, both the incremental and decremental memristor emulator itself incorporates the characteristics of Joglekar's nonlinear ionic-drift model as shown in Figure S2b Therefore, we set the initial value of the memristor emulator of the proposed neuro-memristive synapse to M(0) = 2K and limit its operation within the linear region.

Table S1. Equations of *hp* TiO_2 Memristor and Memristor Emulator.

**Equations of *hp* TiO_2 Memristor**	**Equations of Memristor Emulator [28]**
State-dependent Ohm's law: $v(t) = \left(R_{ON}\frac{w(t)}{D} + R_{OFF}\left(1-\frac{w(t)}{D}\right)\right)i(t)$	State-dependent Ohm's law: $v_{in}(t) = R_s\, i_{in}(t) + v_x(t) = R_s\, i_{in}(t) + \frac{q_{C_T}(t)}{C_T} R_T\, i_{in}(t)$
State Equation: $\frac{dw(t)}{dt} = \mu_V \frac{R_{ON}}{D} i(t)$	State Equation: $\frac{dq_{C_T}(t)}{dt} = C_T \frac{dV_{C_T}(t)}{dt}$
Memristance: $M = \frac{d\varphi}{dq} = R_{OFF}\left\{\left[1+\frac{w_0}{D}\left(\frac{R_{ON}}{R_{OFF}}-1\right)\right] - \mu_V \frac{R_{ON}}{D^2}\left(1-\frac{R_{ON}}{R_{OFF}}\right)q(t)\right\}$ $\approx R_{OFF}\left\{1-\frac{\mu_V R_{ON}}{D^2}q(t)\right\}.$	Memristance of incremental memristor emulator: $M = R_{in} = R_s\left(1+\frac{R_T}{C_T\, R_s}q_{C_T}(t)\right)$ $= R_s + R_T\, v_{C_T}(t), where\ R_s = low\ resistance\ state.$ Memristance of decremental memristor emulator: $M = R_{in} = R_s\left(1-\frac{R_T}{C_T\, R_s}q_{C_T}(t)\right)$ $= R_s - R_T\, v_{C_T}(t), where\ R_s = high\ resistance\ state.$

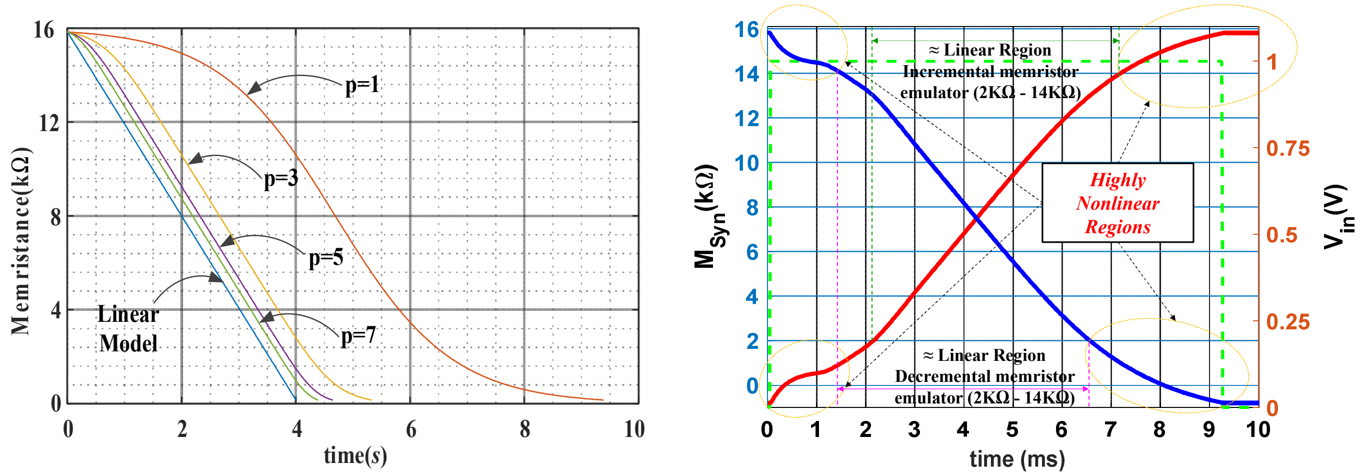

(**a**) (**b**)

Figure S2. (**a**) Relationship between memristance vs. time for the linear and nonlinear models (Joglekar's nonlinear ionic-drift [29]) of the TiO2 memristors, (**b**) memristance vs. time relationship of the incremental and decremental memristor emulator.

The frequency-dependent characteristic (i.e., pinched hysteresis loops) of the memristor emulator is shown in Figure S3. In both bipolar sinusoidal (shown in Figure S3a) and pulse (shown in Figure S3b) inputs, the pinched hysteresis loops pass through the origins. Moreover, the lobe area of the pinched hysteresis loops are decreased with increasing frequency and tends to a singled valued straight line for $f \geq 800$ Hz. Therefore, the memristor emulator, with specified intrinsic parameters as a neuro-memristive synapse, exhibits the defining characteristics of a memristor.

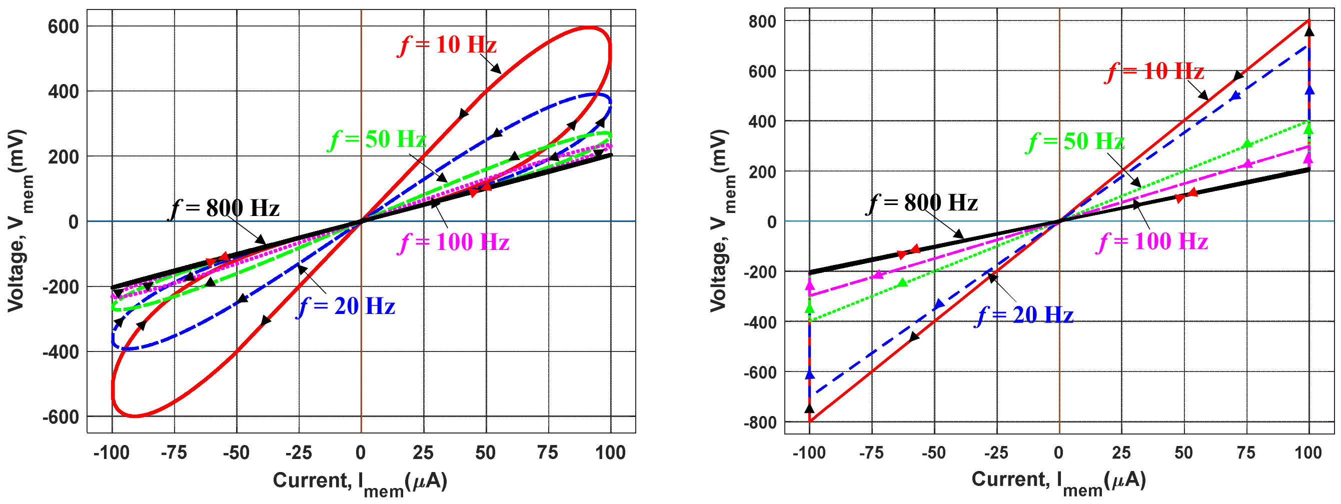

(**a**) (**b**)

Figure S3. Frequency-dependent pinched hysteresis loops for zero-mean periodic stimulation of I_{in} = 100μA for (**a**) bi-polar sinusoidal input, and (**b**) pulse input with frequencies f = 10 Hz, 20 Hz, 50 Hz, 100 Hz, and 800 Hz.

Section 2. Voltage Input Response of Neuro-memristor Synapse

To compare the circuit response for current and voltage input, we stimulated the proposed memristive circuit with a voltage input V_{in} (pulse amplitude PA = 0.75 V, pulse width PW = 1 ms and pulse period PP = 50 ms), as shown in following Figure S4. Observed that the PW and PP of the V_{in} (in Figure S4a), and the V_{MFE} and V_{DEP} (shown in Figure S4b) remain same as that of PW and PP of I_{in} (in Figure 3a), and V_{MFE} and V_{DEP} of Figure 3b of the manuscript. Figure. S4c shows that the current passing through the memristor emulator (I_{mem}) is decreasing with each input pulses. The current I_{mem} (i_{in} in Figure S1) decreases because the potential difference between the nodes of input resistor (R_s) decreases with increasing feedback voltage as shown in Figure S4c. Thus, results in lower current supplies in the capacitor (C_T) and resistor (R_T) (shown in Figure S1). Therefore, the increments in synaptic strength (M_{syn}) for later input cycles are smaller than that of initial input cycles as shown in the inset of Figure S4d. Figure S4e shows that the dissimilar increments in M_{syn} hamper the synaptic voltage accumulations in the later cycle. However, for a current input (i_{in}), the same amplitude and width current is flowing in R_s, and copied to intrinsic capacitor (C) and resistor (R). Therefore, the increment in memristance M_{syn} (i.e., synaptic strength in Figure 3c of the manuscript) for each cycle is almost similar and results in steady distinguishable increments in synaptic voltage (V_{syn} in Figure 3d). The synaptic voltage difference (ΔV_{syn} = 290 mV) of each cycle is sufficient enough to generate the post synaptic firings like as reference [14].

Due to the dissimilar and slower accumulation of M_{syn} (in Figure S4d) in later cycle of a given voltage input, the neuro-memristive synapse might exhibits unintended consequences of erroneous weight updating (for spiking neural networks) or synaptic strength modification (for implementation of bio-realistic attributes). Moreover, it is quite difficult to process such small synaptic voltage difference ($\Delta V_{syn} \approx$ 80 mV) to generate the post synaptic firings like as reference [14].

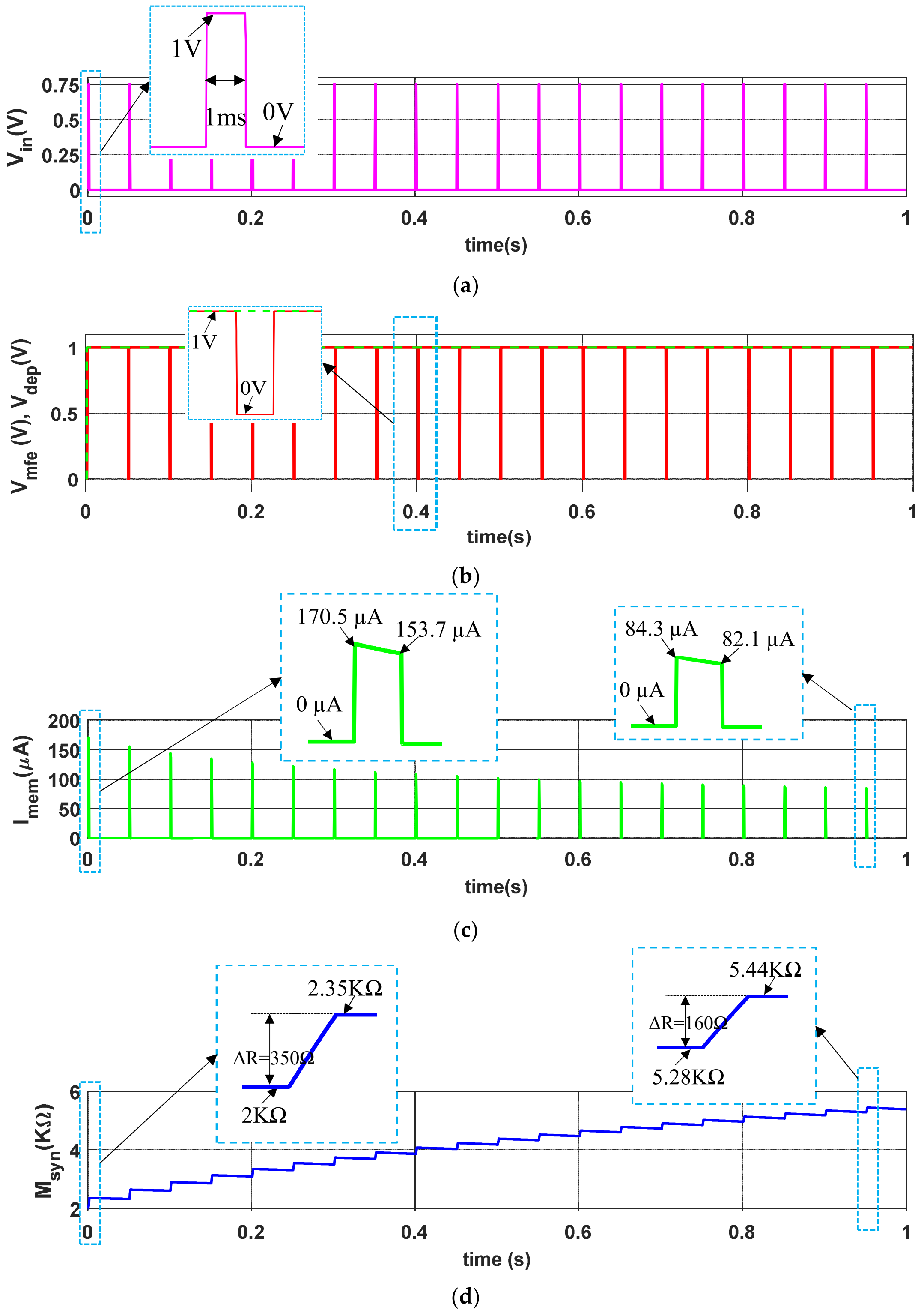
1V
1ms
0V
0.75
0.5
0.25
0
V_{in}(V)
0
0.2
0.4
0.6
0.8
1
time(s)
(a)
1V
0V
1
0.5
0
V_{mfe} (V), V_{dep}(V)
time(s)
(b)
170.5 μA
153.7 μA
0 μA
84.3 μA
82.1 μA
0 μA
200
150
100
50
0
I_{mem}(μA)
time(s)
(c)
2.35KΩ
ΔR=350Ω
2KΩ
5.44KΩ
ΔR=160Ω
5.28KΩ
6
4
2
M_{syn}(KΩ)
time (s)
(d)

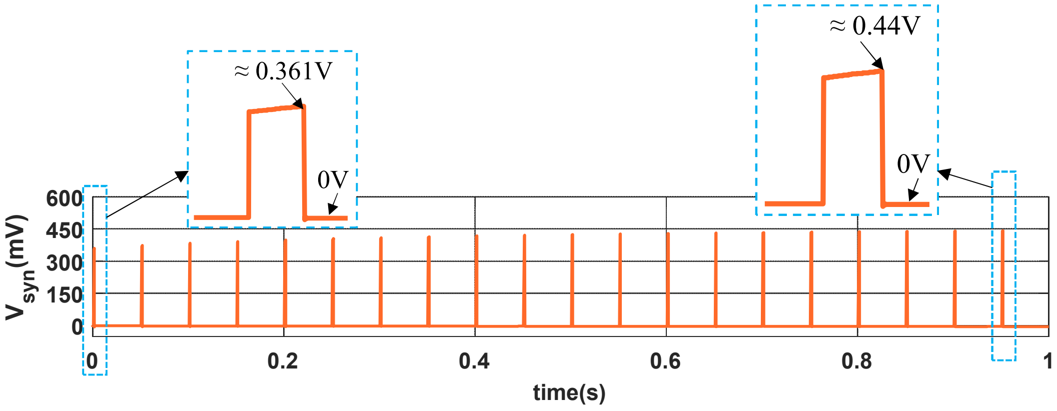

(**e**)

Figure S4. Normal synaptic response of the proposed memristive synapse for a given voltage stimulation: (**a**) voltage stimulation (V_{in} = 0.75 V), (**b**) memory fading effect (V_{mfe}) and depression (V_{DEP}) signals, (**c**) current passing through the memristor emulator of neuro-memristive synapse, (**d**) artificial synaptic strength (M_{syn}), and (**e**) synaptic voltage (V_{syn}).

Therefore, we concluded that the proposed neuro-memristive synapse can operate in voltage mode but it is recommended to operate with current mode to obtain the optimal performance.

Section 3. Shorter and Lengthier Current Input Response of Neuro-memristor Synapse

The pulse duration of biological action potential is typically 1 ms ~ 3 ms. However, most of the neural action potential lasts around 1 ms. Therefore, we chose the pulse width of input stimulation as 1ms.

We stimulated our neuro-memristive synapse with I_{in} (pulse amplitude PA = 100 µA, pulse width PW = 0.25 ms and pulse period PP = 50 ms) where PW is 4 times shorter than the PW in Figure 3 in the main manuscript, and PA and PP remains same as shown in Figure S5a. Figure S5b shows the same V_{MFE} and V_{DEP} signals as in Figure 3 of the manuscript. As expected, the memristive synaptic build-up, shown in the inset of Figure S5c, for shorter pulse width is lesser than the synaptic build-up in Figure 3 (manuscript). Thus, the memristive synaptic voltage, shown in below Figure S5d, of the proposed synapse is smaller than V_{Syn} in Figure 3, and eventually results in the less likelihood of postsynaptic firings.

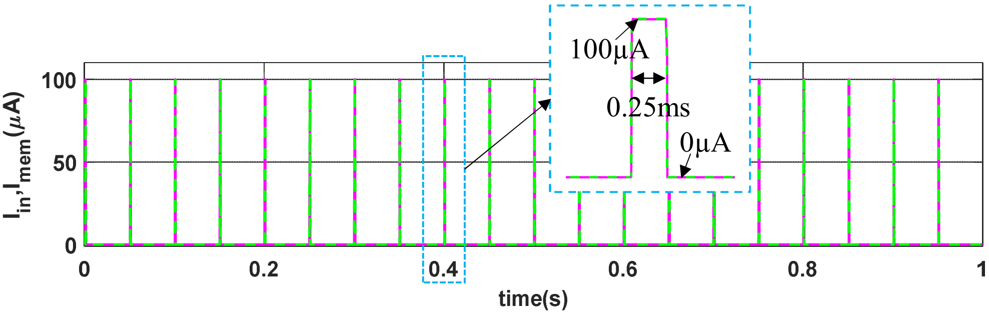

(**a**)

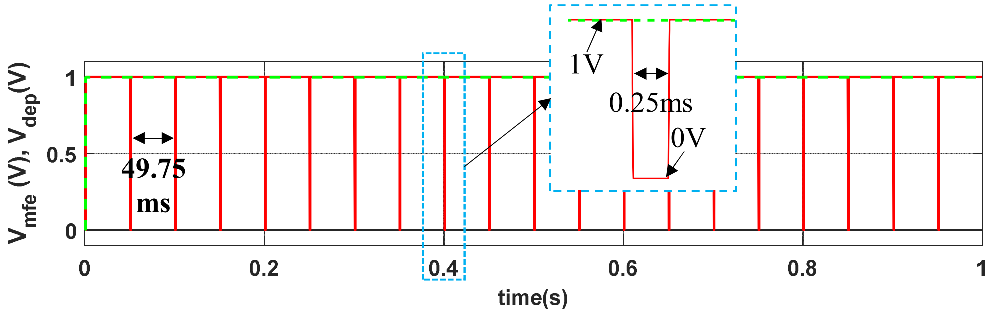

(**b**)

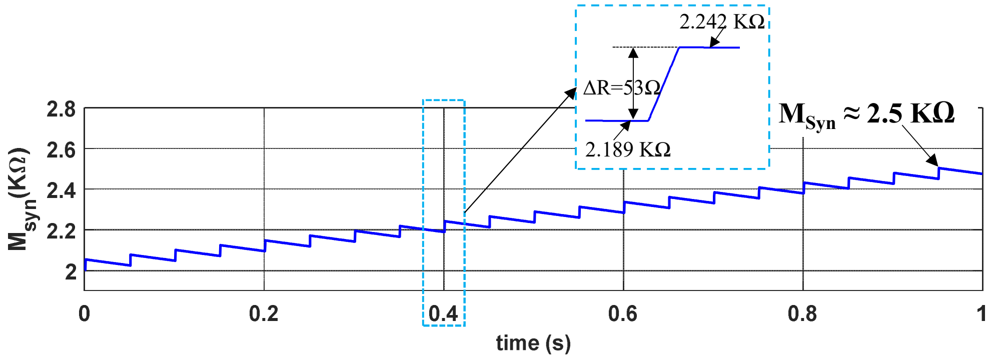

(**c**)

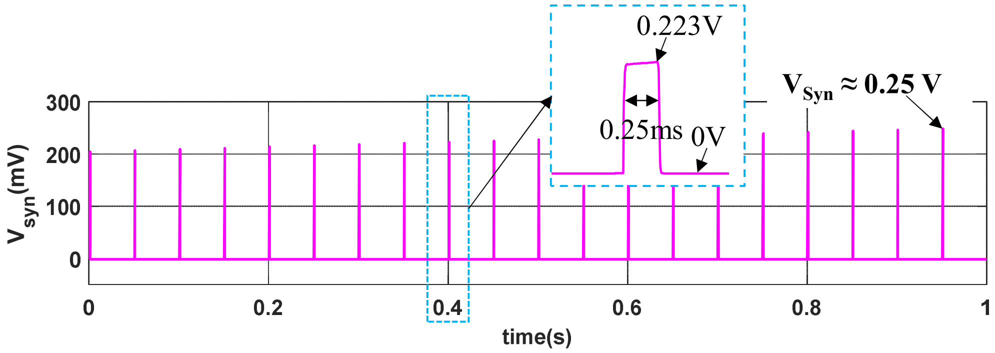

(**d**)

Figure S5. Normal synaptic response of the proposed neuro-memristive synapse with shorter pulse width: (**a**) current stimulus (I_{in}), (**b**) memory fading effect (V_{mfe}) and depression (V_{DEP}) signals, artificial (**c**) synaptic strength (M_{syn}), and (**d**) synaptic voltage (V_{syn}).

We further tested our neuro-memristive synapse with I_{in} (pulse amplitude PA = 100 µA, pulse width PW = 1.5 ms and pulse period PP = 50 ms) where PW is 1.5 times shorter than the PW in Figure 3 (manuscript), and PA and PP remains same, as shown in Figure S6a. Figure S6b shows that the V_{MFE} and V_{DEP} signals are remain same as Figure 3. Expectedly, the memristive synaptic build-up, shown in the inset of Figure S6c, for lengthier pulse width is higher than the synaptic build-up in Figure 3 for which the memristive synaptic voltage, shown in Figure S6c, is higher than V_{Syn} in Figure 3. The higher buildup in synaptic strength and voltage increase the probability of postsynaptic firings.

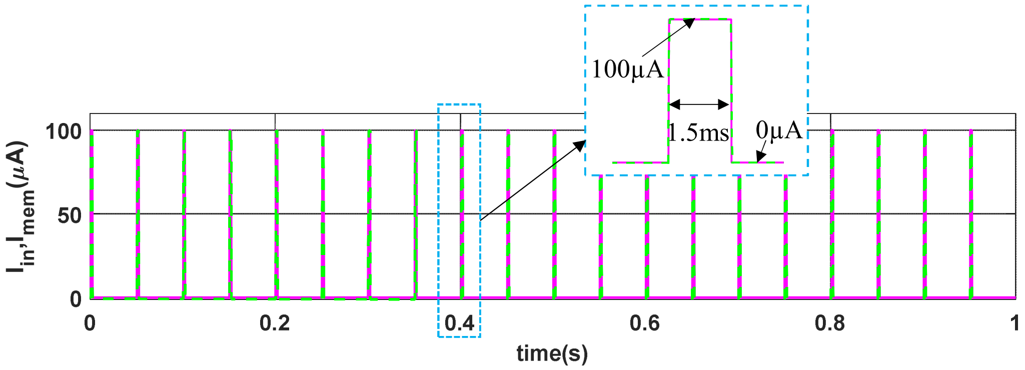

(**a**)

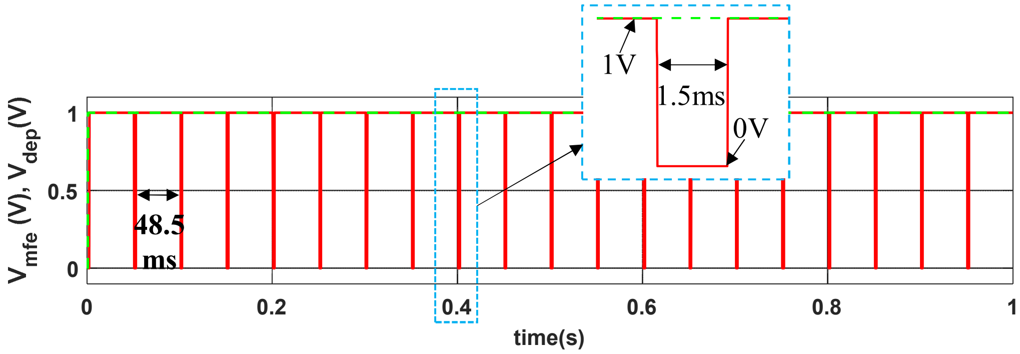

(**b**)

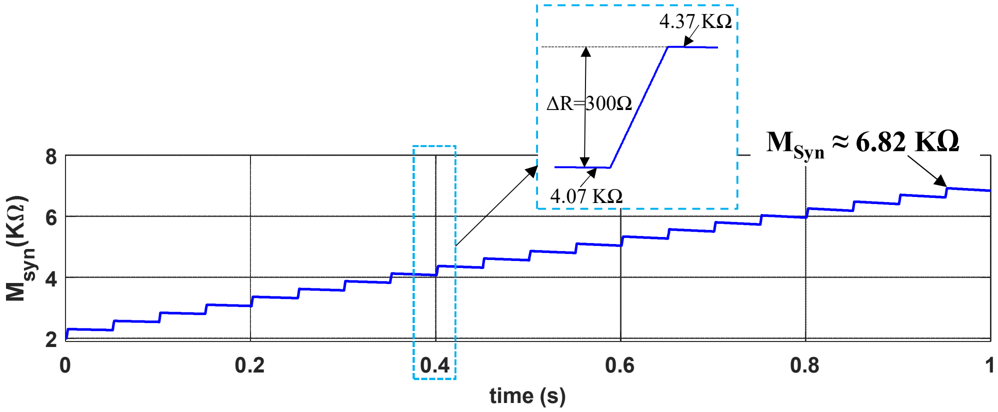

(**c**)

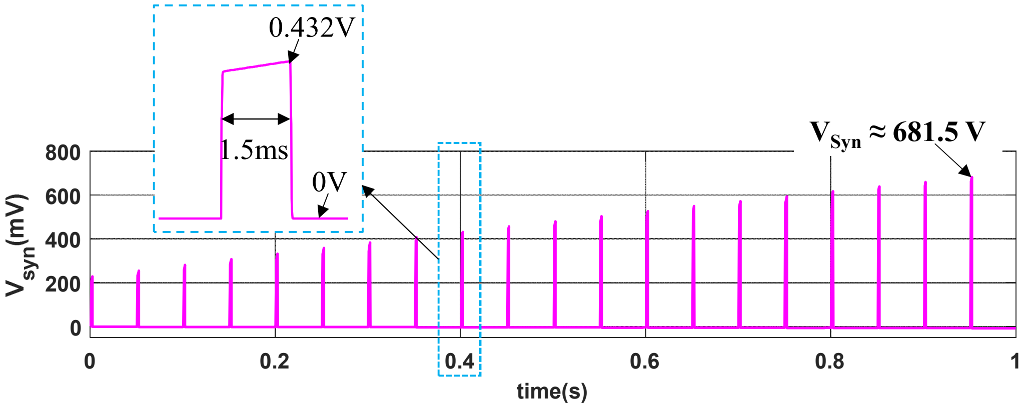

(**d**)

Figure S6. Normal synaptic response of the proposed neuro-memristive synapse with lengthier pulse width: (**a**) current stimulus (I_{in}), (**b**) memory fading effect (V_{mfe}) and depression (V_{DEP}) signals, artificial (**c**) synaptic strength (M_{syn}), and (**d**) synaptic voltage (V_{syn}).
